# Immunoinflammatory mediators in the pathogenesis of *diabetes mellitus*

**DOI:** 10.31744/einstein_journal/2019RB4596

**Published:** 2019-02-19

**Authors:** Bárbara Festa Gomes, Camila de Melo Accardo

**Affiliations:** 1 Faculdade das Américas, São Paulo, SP, Brazil.

**Keywords:** Diabetes mellitus, Insulin resistance, Adipokines, Obesity, Diabetes mellitus, Resistência à insulina, Adipocinas, Obesidade

## Abstract

Characterized as a metabolic syndrome with multiple consequences for the lives of patients, *diabetes mellitus* is also classified as a chronic non-communicable disease of great scope in the world. It is a complex disease, with different points of view, including the relation between inflammatory process, obesity and insulin resistance due to the performance of the various immunoinflammatory mediators - called adipokines - on glycemic homeostasis. Recent studies have precisely addressed this aspect for the development of drugs that assist in the protection of pancreatic ß cells from the damages arising from oxidative stress and inflammatory process, in order to control the hyperglycemic picture, which is characteristic of *diabetes mellitus*.

## RESUMO

Caracterizado como uma síndrome metabólica de múltiplas consequências para a vida de seus portadores, o *diabetes mellitus* é também classificado como uma doença crônica não transmissível de grande abrangência no mundo. Trata-se de uma doença complexa, com diversos pontos de vista, dentre eles a relação entre processo inflamatório, obesidade e resistência à ação da insulina, devido à atuação dos diversos mediadores imunoinflamatórios, chamados de adipocinas, sobre a homeostase glicêmica. Recentes estudos têm abordado justamente este aspecto para o desenvolvimento de fármacos que auxiliem na proteção das células ß pancreáticas dos danos advindos do estresse oxidativo e processo inflamatório, de modo a controlar o quadro hiperglicêmico característico do *diabetes mellitus*.

## INTRODUCTION

It is calculated that approximately 4.6 million deaths occur every year due to the consequences of *diabetes mellitus* (DM).^(^ ^1^ ^)^ According to the International Diabetes Federation (IDF), there are roughly 425 million people with diabetes in the world, and by 2045, this number is expected to grow by roughly 48%, amounting to about 629 million.^(^ ^2^ ^)^

Hyperglycemia is a fundamental characteristic of DM, and results from abnormal functioning of insulin as a hormone regulating glucose metabolism. Insulin-resistance is specifically related to interferences in the signaling process of target-cells.^(^ ^3^ ^)^

It is possible to infer that, by means of a cascade of events, insulin enables glucose transport into the intracellular milieu, by interacting with insulin receptors (IR) present throughout the cell membrane and made up of two α and two β chains (one right after the other, with extra and intracellular domains, respectively). This is how the insulin signaling process starts, promoting successive phosphorylations and activating two major pathways known as mitogen-activated protein kinases (MAPK) and phosphatidylinositol 3-kinase (PI3K), the first acting on insulin-sensitive tissues and the second on glucose and lipid metabolism.^(^ ^4^ ^)^

It is precisely in the pathway of the different post-insulin-signaling reactions that many immunoinflammatory mediators come into place, suppressing outcomes and promoting resistance, which precedes DM *per se*. The objective of this work was to discuss the importance of inflammatory biomarkers in the pathogenesis of DM, and the related scientific novelties. A qualitative search of keywords was carried out, using the keywords “*diabetes mellitus*”, “insulin resistance”, “inflammation”, “adipokines” and “obesity”, at major scientific databases (PubMed^®^, Latin Americana and Caribbean Health Sciences Literature − LILACS − and Scientific Electronic Library Online − SciELO), and at the international health organization related to the subject (the International Diabetes Federation - IDF). The result is provided in this article as a contextualized review and discussion of the data found in animal and in vitro studies about the inflammatory dimension of DM.

## ADIPOSITY AND INFLAMMATORY PROCESS

It is estimated that the prevalence of obese people in the world will grow by approximately 40% until the next decade, rendering great importance to the topic of “obesity” and its multiple and relevant consequences within the public health sphere.^(^ ^5^ ^)^

Excess adipose tissue related to positive caloric balance defines the state of obesity, which is the basis for several metabolic disorders, such as DM. Adipose connective tissue is formed by three types of adipocytes (white, brite and brown), each with specific roles, such as thermoregulation, energy-lipid storage and endocrine function^(^ ^6^ ^)^ - the latter observed in the secretion of immunoinflammatory factors, such as pro-inflammatory cytokines, and factors related with insulin-sensitivity and glucose metabolism, appetite and energy balance, among others.^(^ ^7^ ^)^

With the important endocrine role played by the adipose tissue and the inflammation/obesity/insulin-resistance triad confirmed by the expression of immunoinflammatory markers in tissues (adipocytokines), it is possible to understand the pathogenesis of diabetes and other chronic metabolic diseases. Obesity, especially of the android type, is an important risk factor for the onset not only of DM, but also metabolic syndrome (MS), since it promotes hyperinsulinemia, lipotoxicity and glucotoxicity, which affect the functioning of pancreatic β cells and the whole metabolic homeostasis of the body.^(^ ^8^ ^)^

Also, it is important to stress that adiposity, i.e. hyperplasia and hypertrophy of adipocytes, is intimately linked with increased inflammatory mediators, reduced adiponectin levels, increased insulin-resistance, increased inflammation and hyperinsulinemia, increased oxidative stress and abnormal lipid metabolism.^(^ ^9^ ^)^

The onset of obesity-related type 2 DM is mainly triggered by inflammation of the adipose tissue, which undergoes metabolic changes, such as increased lipolysis, and greater migration and/or differentiation of inflammatory immune cells present in this tissue, including macrophages and T- and B-cells, neutrophils, among others, which contribute to the synthesis of cytokines and adipocytokines. This results in systemic development of insulin resistance.^(^ ^10^ ^)^

## ACTION OF IMMUNOINFLAMMATORY MEDIATORS

Adipocytokines released during chronic low-grade inflammation of the adipose tissue, in the context of obesity, have been directly linked to insulin-resistance and onset of MS and, subsequently, systemic dysfunction and DM.^(^ ^11^ ^)^ Special attention is given to tumor necrosis factor-alpha (TNF-α), resistin and interleukin-6 (IL-6) as immunoinflammatory mediators that promote insulin-resistance. Adiponectin is considered a regulatory adipokine with antagonistic effects associated with vascular protection, increased glucose uptake through better insulin-sensitivity, decreased liver gluconeogenesis, and suppression of pro-inflammatory mediators (IL-6 and TNF-α).^(^ ^12^ ^)^

Immunoinflammatory cytokines IL-6 and TNF-α are excessively secreted in the presence of adipocyte hyperplasia, and macrophage and lymphocyte infiltration into the adipose tissue - by association of the immune system, which slows down the insulin signaling chain and the resulting glucose translocation, and affects energy homeostasis and body mass, contributing to the onset of DM due to insulin-resistance.^(^ ^13^ ^)^

Some studies showed that these mediators also affect regulation of the synthesis of C-reactive protein (CRP), another important inflammatory marker related to tissue injury and cardiovascular events.^(^ ^13^ ^)^ In respect to the connection between the hyperglycemic state resulting from DM and inflammation, it is important to highlight the formation of advanced glycation products, which promote oxidative stress - particularly in visceral fat deposits - and the increased expression of immunoinflammatory mediators, such as IL-6. Moreover, there is activation of macrophages and increased synthesis of reactive oxygen species (ROS), consequently increasing the synthesis of CRP and promoting a proinflammatory state, which evolves to insulin-resistance, MS and DM.^(^ ^14^ ^)^

Hyperglycemia promotes the release of inflammatory mediators and protein glycation, which renders them functionally inactive, as well as auto-oxidation of glucose particles, leading to formation of free radicals and the consequent destruction and dysfunction of certain cells, like the pancreatic β cells that produce insulin. This leads to an important systemic change, which cannot be compensated by the antioxidant system, in charge of eliminating free radicals from the body. The failure of this entire process is known as oxidative stress.^(^ ^15^ ^)^

Another immunoinflammatory mediator featuring in recent studies is interleukin-1 β (IL-1β), which influences the pathogenesis of DM with both its deleterious effects and its physiological role in glucose metabolism. There is an important contribution of IL-1β, which derives from macrophages, in acute postprandial inflammation and postprandial increase of insulin secretion through IL-1 receptors on β cells, whose action depends on glucose and insulin. Both insulin and IL-1β have been seen as glucose level regulators, and IL-1β, in particular, stimulates glucose uptake in immune cells.^(^ ^16^ ^)^

There is also a relevant role of resistin, an immunoinflammatory cytokine in charge of reducing phosphorylations of IRs in their interaction with insulin, by increasing the expression of TNF-α and IL-6, which leads to insulin-resistance and a proinflammatory process, ultimately blocking glucose translocation and promoting a hyperglycemic state. Adipokine visfatin, secreted by adipocytes and macrophages, is another player in insulin sensitization and glucidic metabolism, activating the synthesis of IL-6 and TNF-α.^(^ ^17^ ^)^

**Figure 1:**
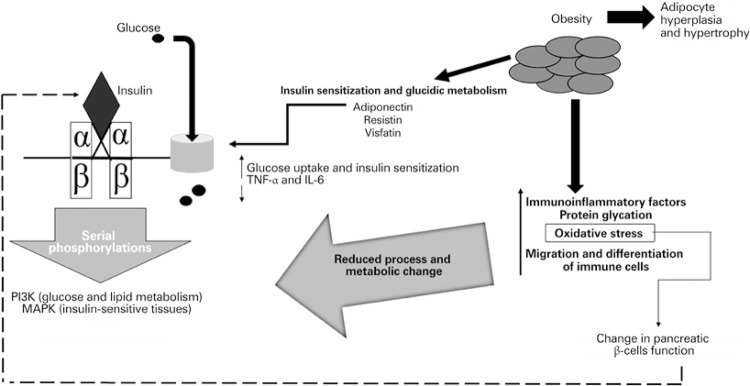
Immune inflammation of the adipose tissue related with *diabetes mellitus* and insulin resistance

Considering this whole context, what Figure 1 shows about obesity-related immunoinflammation and its connection with insulin-resistance is the complexity of how immunoinflammatory mediators are involved in the onset of DM, and the importance of properly identifying the condition to establish the course of evolution of the metabolic imbalance established in the body of diabetic patients.

TNF-α: tumor necrosis factor alpha; IL-6: interleukin 6 (IL-6); PI3K: phosphatidylinositol-3-kinase; MAPK: mitogen-activated protein kinases.

In this scheme, it is possible to see the physiological process of glucose being transferred to the intracellular milieu, through the action of insulin on its specific receptor, triggering serial phosphorylations of molecules that lead to the activation of two pathways, the PI3K (related to glucose and lipid metabolism) and MAPK (related to insulin-sensitive tissues). Obesity also interferes in this process, due to hyperplasia and hypertrophy of adipocytes, which go on to secrete larger amounts of immunoinflammatory factors and promote cell changes, affecting the glucose uptake process and insulin secretion by pancreatic β cells, and leading to DM-related insulin-resistance.

## RECOGNITION OF IMMUNOINFLAMMATORY MEDIATORS ASSOCIATED WITH PROGRESSION AND TREATMENT OF METABOLIC DYSFUNCTION

The diabetic state is defined by clinical examinations, with findings such as polyuria, polydipsia, polyphagia and rapid, spontaneous weight loss, and by laboratory exams, such as fasting glucose, oral glucose tolerance test (OGTT with a 75-gram dextrose challenge) and random glucose.^(^ ^18^ ^)^ It is also interesting to assess DM progression based on the inflammatory process, comparing with body fat distribution, obesity and its risk factors, such as, for instance, cholesterol, as well as glucotoxicity, such as serum insulin and cytokine levels, CRP levels, interleukins and immunoinflammatory markers, in addition to lipid profile, adiponectin levels, and adipose tissue immunohistochemistry, by using methods like Western blot and enzyme-linked immunosorbent assay (ELISA).^(^ ^19^ ^)^ This shows the relevance of our findings, the importance of new research studies on complications resulting from inflammation-related metabolic dysfunctions and, consequently, the need for greater efficacy and more options to manage DM.

In respect to therapies focusing on the inflammatory dimension of DM, clinical trials have been conducted with antioxidant compounds that prevent progression and onset of DM by increasing insulin secretion and protecting β cells against oxidative damage.^(^ ^20^ ^,^ ^21^ ^)^ Recent animal and in vitro studies have shown positive results using drugs that activate the nuclear factor erythroid 2-related factor 2 (Nrf2), increasing protection to β cells against damage resulting from oxidative stress. This prevents DM progression by increasing insulin secretion, improving glycemic control, suppressing proinflammatory cytokines, improving antioxidant and detoxification responses, and regulating metabolism and inflammatory response.^(^ ^22^ ^)^

Nuclear factor erythroid 2-related factor 2 regulates the expression of various genes and is a protective factor to pancreatic β cells, increasing their formation and decreasing their death. It is activated under physiological conditions of elevated glucose levels, but not in a state of glycemic imbalance and, therefore, its production and activation must be externally stimulated so that its role in metabolic homeostasis can be restablished.^(^ ^23^ ^,^ ^24^ ^)^ The use of resveratrol and pterostilbene analogs to promote Nrf2 activation has shown positive results, reducing the level of proinflammatory cytokines in the diabetic state, and maintaining glycemic homeostasis by suppressing inflammatory response and blocking apoptosis of β cells.^(^ ^25^ ^)^

Studies in mice with induced diabetes and human renal mesangial cells using the Nrf2-activating dietary compound sulforaphane showed that this substance may be effective in treating DM and its consequences, such as diabetic nephropathy, by preventing and improving the prognosis of renal injury resulting from the glycemic disorder.^(26)^ These studies may, in the future, after further investigations, be considered as a new therapeutic perspective.

## CONCLUSION

Maintaining glucose homeostasis and controlling the inflammation resulting from adiposity has been the target of many studies for prevention and improvement of *diabetes mellitus* and its multiple complications (diabetic macro- and microangiopathies, nephropathy and retinopathy, and diabetic foot, among others). Thus, the role of adipokines as immunoinflammatory mediators must be further explored when approaching insulin-resistant diabetic patients. The metabolic and immunoinflammatory functions of patients must be holistically investigated. It is important to investigate the levels of inflammatory markers of diabetes, and include in both research and patient management the new drugs developed to suppress inflammation and improve patients’ metabolic profile (oxidative stress, hyperglycemia and other complications). In our research, we found novelties related to *diabetes mellitus* and its connection with inflammation, pointing to advances in our understanding of the pathogenesis and more specific management of this condition. Healthcare professionals must recognize how the entire pathogenesis of *diabetes mellitus* is associated with inflammation, and study the related evidence.
